# hNAG-1 increases lifespan by regulating energy metabolism and insulin/IGF-1/mTOR signaling

**DOI:** 10.18632/aging.100687

**Published:** 2014-08-28

**Authors:** Xingya Wang, Kali Chrysovergis, Justin Kosak, Grace Kissling, Mike Streicker, Glenda Moser, Ruifang Li, Thomas E. Eling

**Affiliations:** ^1^Laboratory of Molecular Carcinogenesis,National Institute of Environmental Health Sciences (NIEHS), Research Triangle Park NC 27709; USA; ^3^Biostatistics Branch, National Institute of Environmental Health Sciences (NIEHS), Research Triangle Park, NC 27709; USA; ^3^Integrated Laboratory Systems, Inc., Morrisville, NC 27560, USA; ^4^College of Pharmaceutical Sciences, Zhejiang Chinese Medical University, Zhejiang, China 310053

**Keywords:** hNAG-1/GDF15, lifespan, metabolism, insulin/IGF-1/mTOR

## Abstract

Nonsteroidal anti-inflammatory drug-activated gene (*NAG-1*) or *GDF15* is a divergent member of the transforming growth factor beta (TGF-β) superfamily and mice expressing *hNAG-1/hGDF15* have been shown to be resistant to HFD-induced obesity and inflammation. This study investigated if hNAG-1 increases lifespan in mice and its potential mechanisms. Here we report that female *hNAG-1* mice had significantly increased both mean and median life spans in two transgenic lines, with a larger difference in life spans in mice on a HFD than on low fat diet. *hNAG-1* mice displayed significantly reduced body and adipose tissue weight, lowered serum IGF-1, insulin and glucose levels, improved insulin sensitivity, and increased oxygen utilization, oxidative metabolism and energy expenditure. Gene expression analysis revealed significant differences in conserved gene pathways that are important regulators of longevity, including *IGF-1*, *p70S6K*, and *PI3K/Akt* signaling cascades. Phosphorylation of major components of IGF-1/mTOR signaling pathway was significantly lower in *hNAG-1*mice. Collectively, hNAG-1 is an important regulator of mammalian longevity and may act as a survival factor. Our study suggests that hNAG-1 has potential therapeutic uses in obesity-related diseases where life span is frequently shorter.

## INTRODUCTION

Aging is characterized by decline in cellular function and is associated with obesity, inflammation, altered energy metabolism, and insulin resistance [1, 2]. Understanding the mechanisms of aging with the goal of increased lifespan remains an area of intensive study. Metabolic dysfunction is a common hallmark of aging [2]. The insulin/IGF-1 (IIS) signaling pathway is the most characterized metabolic pathway implicated in aging [2, 3]. Genetic suppression of IIS signaling extends longevity in worms, insects, and mammals [1]. Caloric restriction is the only efficient treatment known to increase mammalian lifespan other than genetic modifications [4]. In early life, rodents fed a caloric restriction diet have lower IGF-1 levels than rodents fed a normal chow diet, and many rodent genetic models with a prolonged lifespan have lower levels of serum IGF-1 or IIS signaling compared to control groups [5-7]. In contrast, HFD promotes mortality and decreases lifespan in laboratory animals [8, 9].

hNAG-1 plays a complex, but poorly understood role in several human diseases [10]. Circulating hNAG-1 is elevated in physiological and pathological processes, including early pregnancy, liver injury, heart failure, and cancers [11, 12]. hNAG-1 has been shown to play a role in adiposity as transgenic mice expressing hNAG-1 have reduced body weight and fat content compared to their wild-type littermates [2, 13]. Recently, we have shown that *hNAG-1* transgenic mice are resistant to both genetic and dietary-induced obesity, and have improved insulin sensitivity and higher oxidative metabolism compared to wild-type controls [14]. We also found that *hNAG-1* mice have lower inflammation [15, 16]. In addition, *hNAG-1* transgenic mice have significantly reduced serum level of IGF-1[14]. However, it has not been determined if hNAG-1 increases lifespan and if it alters the IGF-1/mTOR pathway, a key pathway in the regulation of aging [4].

In this study, we measured the lifespan of *hNAG-1* transgenic and wild type mice and examined effects of HFD on lifespan. Lifespans of the *hNAG-1* transgenic mice are significantly longer with both LFD and HFD. We also found increased oxidative metabolism, insulin sensitivity, and reduced signaling cascade of IGF-1/Insulin/mTOR in the *hNAG-1* transgenic mice which may be responsible, in part, for the increased lifespan.

## RESULTS

### *hNAG-1* female mice have increased lifespan

**Figure 1:**
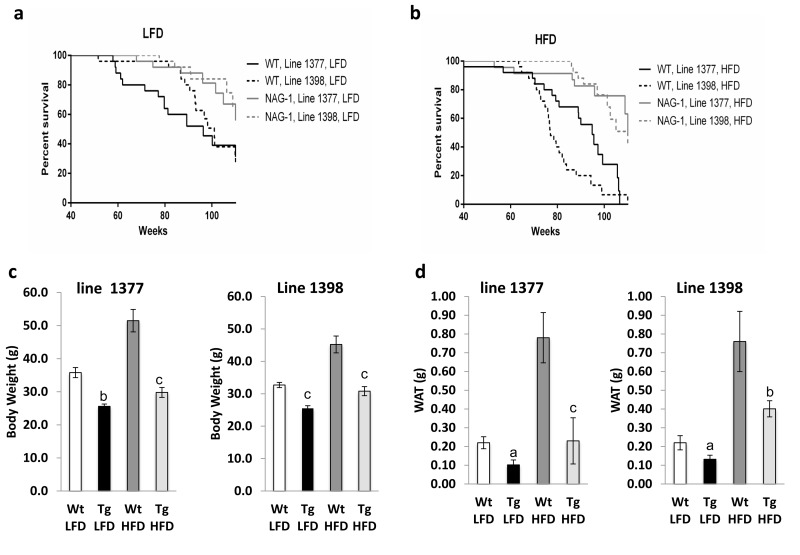
Increased lifespan of female hNAG-1 transgenic mice **(a-b)**, Kaplan-Meier survival curves for female Wt and *hNAG-1* mice from two transgenic lines, line 1377 and line 1398 (**a**) LFD (**b**) HFD. (**c)**, Terminal mean body weights of hNAG-1 and Wt mice at 95±5 wk old in two lines (g). (**d)**, Mean abdominal white adipose tissue (WAT) weights of the mice (g). n=9~18. Data are presented as mean ± SE. a, p<0.05, b, p<0.01 and c, p<0.001 as determined by Student's t-test.

A total of 200 female *hNAG-1* mice and their wild-type (WT) littermates from two transgenic lines (1377 and 1398) were placed on either 10% (LFD) or 60% fat diets (HFD) (n = 25 in each line-genotype-diet group). Log-rank tests showed lifespan was significantly longer for *hNAG-1* mice than for WT mice in both lines with both diets (Figure 1a and 1b, Table 1). On LFD, the median lifespan of hNAG-1 mice was 18.8 weeks (19.5%) and 13.0 weeks (12.8%) longer than WT littermates, respectively for lines 1377 and 1398 (log rank χ^2^ = 4.60, 1 d.f., p=0.032 for line 1377; log rank χ^2^ = 8.03, 1 d.f., p=0.005 for line 1398, Table 1). On HFD, the median lifespan of *hNAG-1* mice was 22.4 weeks (23.6%) and 33.6 weeks (43.6%) longer than WT littermates, respectively for lines 1377 and 1398 (log rank χ^2^ = 13.0, 1 d.f., p<0.001 for line 1377; log rank χ^2^ = 28.9, 1 d.f., p<0.001 for line 1398, Table 1). Consistent with previous findings that HFD could shorten lifespan[8, 9], survival of line 1398 WT mice in the HFD group was significantly lower than the LFD group (median was 24 weeks lower, log rank χ^2^ = 11.2, 1 d.f., p < 0.001). However, there were no significant differences in lifespan between HFD and LFD in either WT or *hNAG-1* transgenic mice in line 1377. Consistent with previous findings that young *hNAG-1*mice (20 to 30 weeks) are resistant to obesity on a 12 week-long HFD [14], both mean body and abdominal white adipose tissue (WAT) weights are significantly reduced in old *hNAG-1* mice (> 95 weeks) after prolonged feeding with either diets (Figure 1c and 1d, Supplementary Figures 1 and 2). With aging and especially on HFD, mice (C57/BL6) develop spontaneous skin lesions and mass growths (tumors or non-tumors) in organs and experience lymph node and spleen enlargement [17-19]. Fewer *hNAG-1* mice have skin lesions, spleen enlargement and gross liver lesions compared to WT littermates (Supplementary Tables 1 and 2). Percentages of mice with lymph node enlargements were similar between *hNAG-1* and WT mice. Collectively, hNAG-1 overexpression increases lifespan in female mice. *hNAG-1* mice are resistant to diet-induced obesity, as well as age- and diet-induced pathological lesions.

**Table 1:** Median survival times (weeks) with comparisons of hNAG-1 and WT mice

		10% Fat Diet (LFD)			60% Fat Diet (HFD)		
Line	Genotype	N	Median	P-Value	N	Median	P-Value
1377	NAG-1	25	115.1	0.032	25	117.3	<0.001
	WT	25	96.3		25	94.9	
1398	NAG-1	25	114.0	0.005	25	110.6	<0.001
	WT	25	101.0		25	77.0	

Median lifespans were calculated by Kaplan–Meier survival curve analysis. Statistical comparisons between genotypes and diets was performed using the log rank test (N=25, p<0.05).

### *hNAG-1* female mice have increased circulating growth hormone level

**Figure 2:**
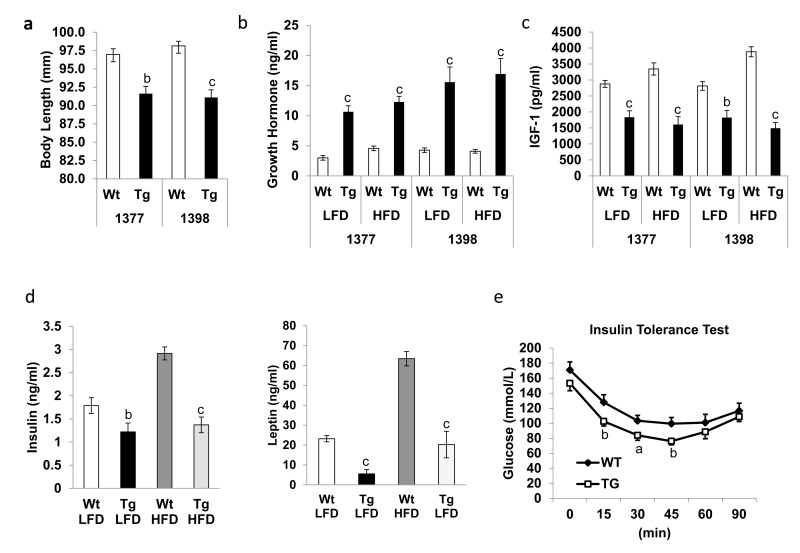
*hNAG-1* transgenic mice have improved insulin sensitivity **(a),** Mean body length of female hNAG-1 and Wt mice in both lines (n=6~8). **(b-d)**, Mean serum levels of growth hormone (**b**), IGF-1 (**c**), insulin and leptin (**d**) in terminal blood of hNAG-1 and Wt mice on LFD and HFD (n=10). **e**, Insulin tolerance test on old (>95 wk) 1398 female hNAG-1 Tg mice and Wt mice (n=8~9). Data are presented as mean ±SE. a, p<0.05, b, p<0.01 and c, p<0.001 as determined by Student's t-test.

In mice, disruption of growth hormone (GH) signaling that leads to major alterations in IIS has a well-documented positive impact on lifespan [4]. The long-lived GH-resistant GH receptor (GHR)-KO mice which have high GH level, as well as Ames dwarf and Snell dwarf mice lacking GHexhibit dwarfism, increased subcutaneous adiposity, change in tissue sizes, and increased insulin sensitivity[1, 20]. Although *hNAG-1* mice are significantly shorter in body length (Figure 2a) there are no significant differences in tissue sizes, length of femurs or organ weights (data not shown). Interestingly, circulating GH level is substantially increased in terminal serum of *hNAG-1* mice (Figure 2b). This difference is more dramatic in young 1398 female mice (Supplementary Figure 3) suggesting a possible GH-resistant phenotype or simply a feed-back regulation due to reduced IGF-1 level in *hNAG-1* mice (Figure 2c). However, the expression of GHR in liver, WAT, or brain tissue at both mRNA and protein levels was the same for *hNAG-1* and WT mice (data not shown). The downstream GH signaling cascade including phosphorylation of JAK2, ERK1/2, and SMAD were not different in *hNAG-1* mice. Recombinant hNAG-1 did not inhibit GH-induced downstream signaling or IGF-1 secretion in cell culture studies using HepG2 cells (Data not shown). These data suggest that *hNAG-1* mice are not GH resistant and the effects on longevity in *hNAG-1* mice appear to be independent of GH. However, the cause of the reduction in IGF-1 levels and elevated GH levels in *hNAG-1* mice is unclear.

### *hNAG-1* female mice have increased insulin sensitivity

The resistance against HFD-induced obesity suggests that the transgenic expression of *hNAG-1* in mice might positively affect age-associated metabolic disorders, such as insulin resistance. Similar to young mice [16], insulin and leptin levels are substantially reduced in *hNAG-1* mice on LFD or HFD in old mice (Figure 2d).

Insulin tolerance test (ITT) shows that 95week-old (line 1398) *hNAG-1* mice have significantly improved insulin sensitivity compared to WT littermates (Figure 2e). Because the body weights between *hNAG-1* mice and the WT mice are quite different, it was not possible to match same weight at the same age. We decided to use the mice at the same age, which we think is more appropriate than weight. We also calculated the results using % of basal glucose level and found similar results as using glucose concentration (data not shown). Basal glucose levels are also significantly lower in 95 week-old *hNAG-1* transgenic mice. Thus, consistent with association between improved insulin sensitivity with better survival [4], older *hNAG-1* mice sustain higher insulin sensitivity which may play a role, in part, for the increased lifespan as observed above. *hNAG-1* mice mimic caloric restriction with reduced circulating IGF-1 levels, improved insulin sensitivity, and extended lifespan. There is no significant difference in food intake between the mice on a LFD or HFD in both lines (Supplementary Figure 4 and 5).

### *hNAG-1* have increased energy expenditure and metabolism

**Figure 3:**
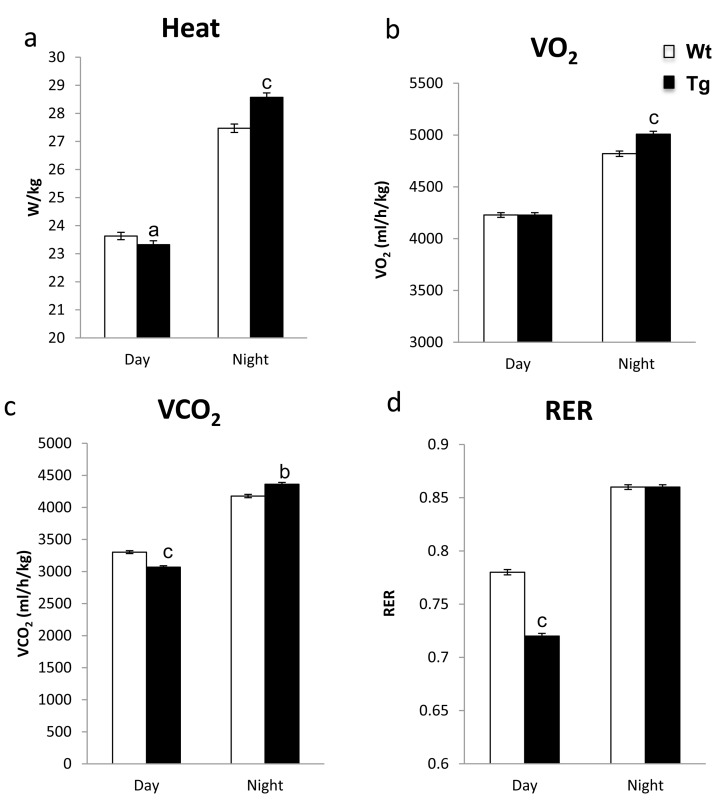
*hNAG-1* transgenic mice have increased metabolism **(a),** Heat production (W/kg) in female *hNAG-1* and Wt mice as determined indirect colorimeter (n=5). (**b-c)**, Consumption of O_2_ and VO_2_ (ml/h/kg) in female hNAG-1 and Wt mice during day and night time (n=5). (**d)**, Respiratory quotient (RER) as calculated from VO_2_ and VCO_2_ in 1398 *hNAG-1* female mice. Data are presented as mean ± SE. a, p<0.05, b, p<0.01 and c, p<0.001 as determined by Student's t-test.

Increased energy expenditure can extend lifespan and alter insulin sensitivity and resistance to obesity [21, 22]. Previously, we have shown that hNAG-1 expression increases metabolic oxidation and thermogenesis in male mice which is responsible, in part, for the resistance of *hNAG-1* mice to obesity [14]. Enhanced O_2_ consumption and higher heat expenditure are consistent with increased oxidative metabolism and is associated with protection from HFD-induced obesity and enhanced longevity [1, 23]. In this study, heat production is slightly but statistically significant lower during the day in female line 1398 hNAG-1 mice (Figure 3a). During the night, *hNAG-1* mice have significantly much higher heat production than WT littermates (Figure 3a) with no difference in physical activity between the mice (data not shown). We then examined the expression of representative thermo-genesis genes in brown adipose tissue (BAT) in both 30 wk and 95 wk old mice. However, unlike the young male mice where most of these genes (*UCP-1, PGC1a, ECH-1, Cox8b, PGC1b*, etc) are up regulated in BAT [14], only few genes were up regulated in BAT in female mice due to larger variation (Supplementary Figure 6). In addition, *hNAG-1* mice utilize more oxygen than WT mice during the night (Figure 3b) and have reduced respiratory quotient (RQ) during the day (Figure 3d), implying greater reliance on fats, as opposed to carbohydrates, as an energy source.

### *hNAG-1* female mice have decreased mTOR signaling activity

**Figure 4:**
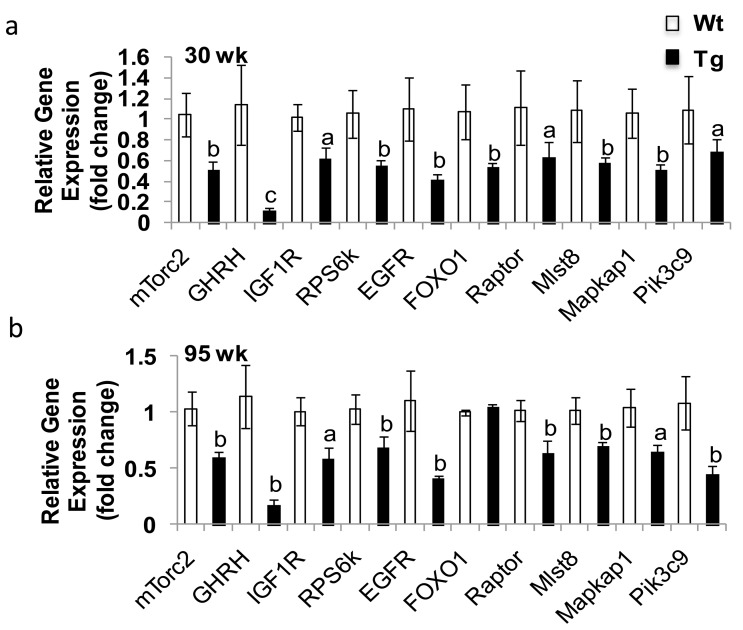
Gene validation from microarray results Validation was analyzed by qRT-PCR. **(a-b),** Validation of the expression of down-regulated genes from microarray study in young (30 wk, **a**) and old (95 wk, **b**) abdominal white adipose tissue (WAT) of female mice (n=6). Data are presented as mean ± SE. a, p<0.05, b, p<0.01 and c, p<0.001 as determined by Student's t-test.

To further understand the mechanism of extended lifespan in *hNAG-1* mice, we used whole genome microarray analysis to examine differential gene expression in abdominal WAT in line 1398 *hNAG-1* mice. Differential category expression analysis showed significant differences between *hNAG-1* mice and WT mice in key pathways in the regulation of metabolism and mammalian lifespan, including down regulation of IGF-1, PI3K, AMPK, and RPS6K families (Supplementary Fig. 7, Table 2). We validated a set of genes (*mTorc2*, *GHRH*, *IGF1R*, *RPS6k*, *FOXO1*, *Raptor*, *Mlst8*, *Mapkap1*, *Pik3c9*) by *qRT-PCR* in young female mice (Figure 4a). The expression pattern of all of these genes confirmed the microarray data with a similar expression pattern in old mice (> 95 wk) except for *FOXO1* (Figure 4b).

**Table 2:** Ingenuity canonical pathways enriched by genes differentially expressed in hNAG-1 mice

Ingenuity Canonical Pathways	Molecules
**Significantly changed**	
PTEN Signaling	RPS6KB1,FOXO1,MAPK1,BMPR1A,FGFR1,IGF1R,MRAS,ITGA5,KRAS,EGFR
IGF-1 Signaling	RPS6KB1,NEDD4,FOXO1,MAPK1,IGF1R,SRF,MRAS,KRAS
p70S6K Signaling	GNAI2,RPS6KB1,F2RL1,MAPK1,MRAS,PRKCE,KRAS,PPP2R5A,EGFR
ErbB Signaling	RPS6KB1,FOXO1,MAPK1,MRAS,PRKCE,KRAS,EGFR
AMPK Signaling	RPS6KB1,CHRNA4,ACACB,MAPK1,MRAS,ACACA,CHRNE,AK2,PPP2R5A
Growth Hormone Signaling	RPS6KB1,MAPK1,IGF1R,SRF,RPS6KA3,PRKCE
PI3K/AKT Signaling	RPS6KB1,FOXO1,MAPK1,MRAS,ITGA5,KRAS,PPP2R5A,THEM4
ERK/MAPK Signaling	BRAF,MAPK1,PLA2G4C,SRF,MRAS,PRKCE,ITGA5,TLN1,KRAS,PPP2R5A
**Non-Significantly changed**	
HIF1α Signaling	SLC2A5,MAPK1,Vegfb,MRAS,EGLN3,KRAS
EGF Signaling	RPS6KB1,MAPK1,SRF,EGFR
ErbB2-ErbB3 Signaling	FOXO1,MAPK1,MRAS,KRAS
mTOR Signaling	RPS6KB1,RND3,MAPK1,Vegfb,MRAS,RPS6KA3,PRKCE,KRAS,PPP2R5A
ErbB4 Signaling	MAPK1,MRAS,PRKCE,KRAS
Role of JAK1 and JAK3 in γc Cytokine Signaling	MAPK1,SH2B2,MRAS,KRAS
Regulation of eIF4 and p70S6K Signaling	RPS6KB1,MAPK1,AGO3,MRAS,ITGA5,KRAS,PPP2R5A
Insulin Receptor Signaling	RPS6KB1,FOXO1,MAPK1,SH2B2,MRAS,KRAS
JAK/Stat Signaling	MAPK1,MRAS,KRAS
PI3K Signaling in B Lymphocytes	MAPK1,SH2B2,MRAS,KRAS

IPA analysis was used to generate a list of genes changed in canonical pathway. Significance was calculated

mTOR pathway is emerging as a key regulator of aging, and is linked to IIS pathway [7, 24]. Inhibition of mTOR activity or knockout the downstream effector of mTOR, S6K1, increases lifespan in mice [25, 26].

**Figure 5:**
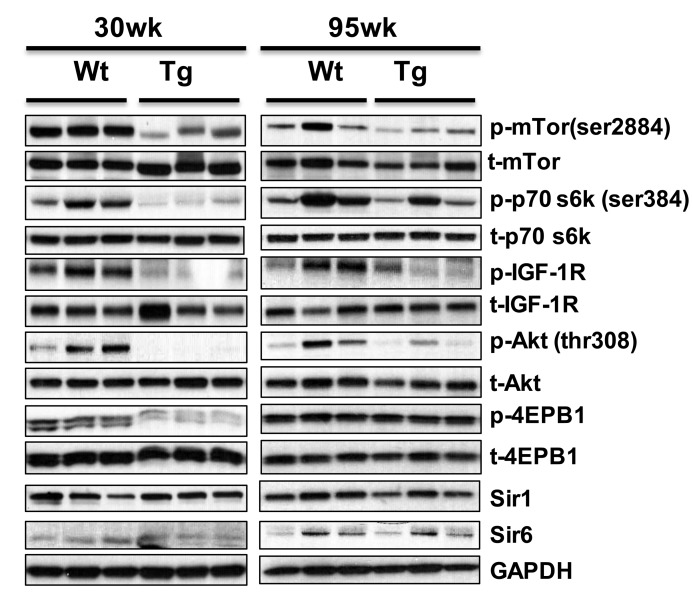
*hNAG-1* transgenic mice have reduced IGF-1/Insulin/mTOR signaling Phosphorylation of proteins of IGF-1/insulin/mTOR signaling pathway was analyzed by Western blot (n=3) in abdominal WAT. Both samples from 30 wk and 95 wk old animals were analyzed.

Phosphorylation of members of mTOR signaling pathway (IGF1R, mTOR (ser2448), AKT (ser308), p70S6k (ser 384), and 4EPB-1) is down regulated in the WAT of young 1398 *hNAG-1* mice (Fig. 3f), and similar pattern is observed in old mice (> 95 wk, Figure 5). The expression of Sirt1 and Sirt6 [6, 27] between *hNAG-1* and WT mice was not different in liver (data not shown) and abdominal WAT (Figure 5).

## DISCUSSION

Aging is characterized by decline in cellular function and is associated with obesity, inflammation, energy metabolism, and insulin resistance [1, 2]. In this study, we report that hNAG-1 is a novel regulator of mammalian longevity. Both the median and mean lifespan of the female *hNAG-1* mice on HFD and LFD are significantly longer than the WT mice. In the male *hNAG-1* transgenic mice, elevated circulating serum levels of hNAG-1 increases thermogenesis and oxidative metabolism [14], inhibits inflammation [15] and improves glucose tolerance [14]. In this study, we also found that female *hNAG-1* mice have increased metabolism and insulin sensitivity.

**Figure 6:**
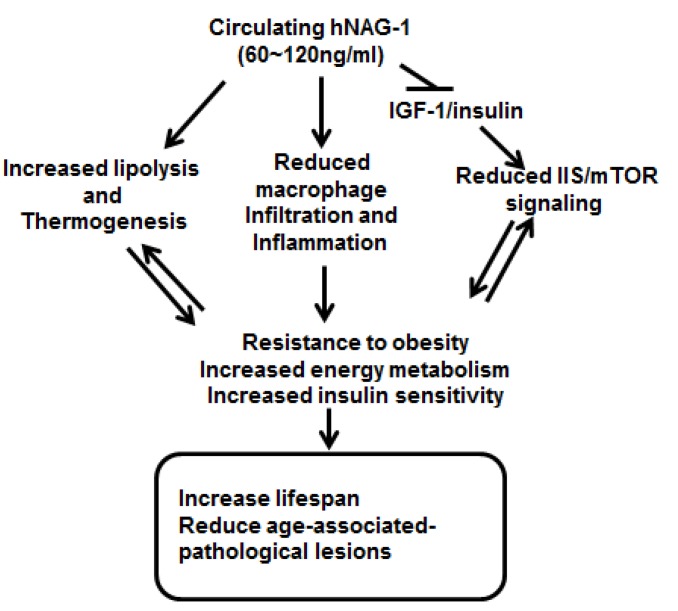
Schematic model for increased survival and lifespan in *hNAG-1* mice Overexpression of hNAG-1 in female mice lowers serum levels of IGF-1 and insulin and thus reduces IGF-1/insulin (IIS)/mTOR signaling. Circulating hNAG-1 also increases lipolysis, thermogenesis, and metabolism in *hNAG-1* mice [14], and reduces macrophage infiltration into WAT and reduces inflammation in *hNAG-1* mice [15, 16]. *hNAG-1* mice are thus resistant to obesity, have increased energy metabolism and increased insulin sensitivity, which leads to increased lifespan, and reduced age- or dietary-induced pathological lesions.

Furthermore, differential gene expression analysis revealed significant differences in the expression of key pathways associated with metabolism and mammalian lifespan between the *hNAG-1* mice and wild-type littermates. Our study suggests that *hNAG-1* improves mammalian survival by increasing energy metabolism and reducing IGF-1/mTOR signaling, two well-studied and tightly regulated longevity networks [4]. These biochemical events are conserved across species and are associated with increased longevity in all organisms and thus it can be assumed they have a role in the increased lifespan in female *hNAG-1* mice as summarized in Figure 6. The *hNAG-1* mice have most of the metabolic characteristic of long-lived mice [1]. In addition, we have previously reported that the *hNAG-1* mice have a lower inflammatory response [15, 16], and inflammation has been associated with aging [28, 29]. In contrast to most long-lived animals in which genetic deletion increases life span, we shown here that an increase in the expression of hNAG-1 protein extended longevity. hNAG-1 has similar effects as observed in Klotho transgenic mice in which lifespan has also been extended by perturbing IGF-1 signaling [30]. hNAG-1 is a circulating protein and thus there are opportunities and ways to alter metabolic activity and influence longevity by increase the circulating levels of hNAG-1 in humans. This is the first report showing hNAG-1 can act to increase lifespan in mice.

In laboratory mice, mutations with GH or GHR have been well documented to have positive impact on lifespan [1]. These mutations lead to alterations in IIS cascade and downstream signaling pathways. In this study, we found that GH is substantially up-regulated in *hNAG-1* mice. Notably, GHR deficient mice also have significantly increased GH level which causes GH resistance [20]. As expected, circulating IGF-1 levels are significantly reduced in old female mice. In line with our findings, IGF-1 deficient mice also produce elevated levels of GH [31], suggesting a possible similarity of growth hormone resistance between hNAG-1 mice and GHR deficient mice. However, we did not detect any differences of GHR expression at mRNA and protein levels between *hNAG-1* and WT mice in liver, brain, and adipose tissues. In addition, *in vitro* cell culture studies also failed to prove hNAG-1 may have inhibitory effects on GH downstream signaling or IGF-1 secretion from cells upon GH treatment. Interestingly, a striking difference between GHR deficient or GH disrupted mice and *hNAG-1* mice is that GHR deficient mice are rather obese which have increased subcutaneous adiposity while *hNAG-1* mice have decreased adiposity [1]. These data suggest that the mechanisms how IGF-1 level is significantly reduced and why GH levels is elevated in hNAG-1 mice is still unclear. *hNAG-1* mice may have both an overlapping and different mechanisms with GH deficient and resistant animals that have a significant effect on extending lifespan.

Female IGF1R heterozygous knockout mice live on average 33% longer than their wild-type littermates, suggesting IGF-1 receptor may be a central regulator of mammalian lifespan [5]. In line with this observation, the expression of *IGFR* at mRNA level is significantly reduced in adipose tissue of *hNAG-1* mice then WT mice. In addition, the phosphorylation of IGF1R is also significantly reduced in *hNAG-1* mice compared to wild-type littermates. The nutrient sensing mTOR pathway is emerging as a key regulator of ageing. mTOR signaling complex affecting several crucial cellular functions, which show clear effects on ageing [24]. mTOR activity is linked to IIS pathway through multiple connections. Inhibition of mTOR by rapamycin or knockout the downstream effector of mTOR, the S6K1 (Rps6k), were reported to increase lifespan in mice [26]. Both microarray data and validation studies suggest that the key family of mTOR pathway and its downstream signaling are downregulated in *hNAG-1* mice. These findings are consistent with studies showing reduced phosphorylation of insulin/IGF-1/mTOR signaling pathway in WAT which is positively linked with prolonged lifespan [24].

The expression of hNAG-1 is significantly higher in many physical conditions and diseases in humans [11, 32]. The serum level of hNAG-1 is higher in patients with cancer, after cardiovascular incidence for example a heart attack, and liver or lung injuries, but is also higher during pregnancy [11, 32]. Serum levels of hNAG-1 have been proposed as a marker for all-cause mortality with concentrations correlated with survival time [33]. These apparent contradictory findings may point to pleiotropic/diverse functions of hNAG-1 in many diseases and physiological conditions. However, a rational explanation for these observations is that hNAG-1 expression is increased by the cellular injury and is acting as a survival factor. Thus, an increase in circulating levels of mature hNAG-1 observed, with the level assumed to be directly related to the severity of the disease/injury. *hNAG-1* mice are protected against chemical and genetically induced intestinal cancers and have lower inflammatory response [15, 34], while the circulating level of hNAG-1 is higher in cancer patients. Likewise, other evidences from mice experiments indicate that hNAG-1 protects the heart from ischemia/reperfusion [31] and hypertrophic injury [12] while circulating levels of hNAG-1 are increased after a cardiovascular event. In cancer patients the increased circulating hNAG-1 is related to the weight loss/cachexia observed [28, 29]. These seemingly conflicting results are consistent with the hypothesis that hNAG-1 acts as a survival factor.

Our recent studies confirmed that raising the circulating levels of the secreted mature hNAG-1 increases thermogenesis and oxidative metabolism causing a dramatic reduction in body and fat contents [14]. hNAG-1 exists in many forms, the pro-monomer, the pro-dimeric form and the secreted dimeric protein is present in the circulation, but the biological activity of the different hNAG-1forms is poorly understood [30]. The conflicting and contradictory findings may be related to some differences in the biological activities of the forms of hNAG-1. In addition, the receptor(s) of hNAG-1 has not been characterized and thus some difference in findings could be related to differences or mutation in these uncharacterized receptors.

In summary, hNAG-1 increased longevity and lifespan in female transgenic mice expressing the human hNAG-1 protein. hNAG-1 increases oxidative metabolism, lower obesity and decrease the insulin/IGF-1 pathway, all of which are associated with survival and longevity. These findings suggest hNAG-1 is a possible novel protein with potential therapeutic uses in diseases such as diabetes and obesity where shorter life span frequently is observed.

## METHODS

### Animals

The *hNAG-1* transgenic mice were generated as described previously [34]. hNAG-1 is expressed in most tissues, liver is very low but good expression is found in the skin, colon, kidney, brain and is highly expressed in the WAT and BAT (data not shown). Experiments were performed in accordance with the “NIH Guidelines for the Use and Care of Laboratory Animals”. A total of 200 WT and *hNAG-1* female transgenic mice at 50 wk old from line 1377 and 1398 were randomized and fed LFD or HFD (Research Diet) and water *ad libitum* (n=25/group). A total of 200 male mice were initially included for longevity study. However, due to relative high percentage of male *hNAG-1* mice that develop, for unknown reasons, urinary blockage which can cause early death the male mice were removed from study. Food intake was measured once a week for 40 weeks. Body weights were measured twice weekly over the course of the study. The mice were euthanized and total fat was removed and weighed. Physical activity, VO_2_, VCO_2_, and body heat production were measured at 30 wk of age in line 1398 female mice as described previously [35].

### Insulin Tolerance Test

Female mice, 95 wk old (1398), were fasted for 6 h and insulin (Sigma, St. Louis, MO) was intraperitoneally injected into mice at dose of 0.5 U/kg BW. Blood was collected from the tail at 0, 15, 30, 60, and 90 min after injection and glucose concentration was analyzed using a glucose meter.

### Microarray Analysis

RNA samples from abdominal WAT of line 1398 female NAG-1 mice and WT mice at 30 wk old were extracted as described [16]. Sample hybridization was performed as previously described [36]. Microarray analysis was performed using Affymetrix Mouse Genome 430 2.0 Array by Microarray core at NIEHS. Pathway analysis was carried out using Ingenuity Pathway Analysis (IPA, http://www.Ingenuity.com). Separate statistical analyses were conducted for each transgenic line.

### Real-time PCR

RNA samples from abdominal WAT and BAT of line 1398 female NAG-1 mice and WT mice at 30 wk old were extracted as described[16]. One microgram of RNA was reverse transcribed using iScript cDNA synthesis kit from BioRad. Real-time PCR assays were performed using Taqman master mix and primers (Applied Biosystems, Foster City, CA) by MyiQ PCR detection system (BioRad) for semi-quatitative real-time PCR analysis. Relative fold changes were calculated using the delta delta Ct method, with β-actin or GAPDH serving as control genes.

### ELISA Analysis

Terminal bleeds from mice were incubated at room temperature for 1 hour in serum separator tubes (Sarstedt, Nümbrecht, Germany) and then spun at 10,000 × rpm for 5 minutes. Serum was collected and stored at −80°C until analysis. Mouse leptin, mouse growth hormone, human NAG-1, mouse IGF-1 (R&D, Minneapolis, MN) and mouse insulin (Alpco, Salem, NH) ELISA kits were used according to manufacturer's instructions.

### Western Blots

Western Blots were performed as described[16]. Total protein in abdominal WAT was isolated using RIPA buffer (Pierce, Rockford, IL) supplemented with sodium fluoride and sodium vanadate. A total of 45 μg protein was electrophoresed on a 4-15% SDS-polyacrylamide Tris-Hcl gel (Bio-Rad) at 175V for 1 h. After transfer, membranes were blocked with 5% non-fat dry milk in 1x TBST (50Mm Tris pH7.5, 150 mM NaCl, 0.1% Tween-20) at room temperature for 1 hour. The membrane was probed overnight at 4°C with appropriate antibodies, at the dilutions as recommended by their manufactures. The next day, blots were rinsed and probed with the appropriate horseradish-peroxidase secondary antibody for 1 hour at room temperature in 5% milk in TBST and illuminated with Western Lightening TM Plus-ECL Enhanced Chemiluminescence Substrate assay kit. The membrane was stripped using Restore Western Blot Stripping Buffer according to manufacturer's instruction. After stripping, the membrane was re-probed for GAPDH for loading control.

### Statistical Analysis

Data were analyzed by Dr. Grace Kissling of the NIEHS Biostatistics Branch who determined the appropriate method to use. Survival was compared between genotypes and diets using log-rank tests; survival curves were plotted from Kaplan-Meier estimates. Body weight was compared between genotypes and diets using polynomial growth curve analysis. Food consumption was compared between genotypes and diets using Mann-Whitney tests with Bonferroni correction for multiple testing. All p-values are two-sided and considered significant at the 0.05 level. Body weight and food consumption plots show mean ± standard error of the mean.

## SUPPLEMENTARY DATA FIGURES AND TABLES


