# Supplementary material for: hNAG-1 increases lifespan by regulating energy metabolism and insulin/IGF-1/mTOR signaling
Source: Aging (Albany NY). 2014 Aug 28;6(8):690–704. doi: 10.18632/aging.100687 (PMC4169862; doi:10.18632/aging.100687)
Supplement: Supplementary file 1 [file aging-06-690-s001.pdf]

SUPPLEMENTARY DATA

Supplementary Table 1. Mean number of lesions in *hNAG-1* and WT mice on LFD and HFD. At necropsy, lymph nodes enlargement, spleen enlargement, liver gross, skin lesions and other masses were recorded.

Line	Diet	Geno-type	N	Gross findings	Lymph nodes enlarged	EnlargeSpleen	Liver gross findings	Skin Lesions	Masses
1377	LFD	Wt	23	4	6	7	9	9	5
1377	HFD	hNAG-1	23	6	6	4	6	5	2
1377	LFD	Wt	24	4	7	7	11	8	4
1377	HFD	hNAG-1	25	6	9	3	3	3	2
1398	LFD	Wt	25	1	12	10	8	17	7
1398	HFD	hNAG-1	25	9	6	3	9	4	4
1398	LFD	Wt	25	2	6	9	10	16	3
1398	HFD	hNAG-1	25	2	8	4	5	8	4

Supplementary Table 2. Incidence of gross lesions in *hNAG-1* and WT mice on LFD and HFD. Percentage incidence of gross lesions was calculated as number of lesions/total number of mice in each study group.

Line	Diet	Geno-type	N	Gross findings	Lymph nodes enlarged	EnlargeSp leen	Liver gross findings	Skin Lesions	Masses
1377	LFD	Wt	23	17.4	26.1	30.4	39.1	39.1	21.7
1377	HFD	hNAG-1	23	26.1	26.1	17.4	26.1	21.7	8.7
1377	LFD	Wt	24	16.7	29.2	29.2	45.8	33.3	16.7
1377	HFD	hNAG-1	25	24.0	36.0	12.0	12.0	12.0	8.0
1398	LFD	Wt	25	4.0	48.0	40.0	32.0	68.0	28.0
1398	HFD	hNAG-1	25	36.0	24.0	12.0	36.0	16.0	16.0
1398	LFD	Wt	25	8.0	24.0	36.0	40.0	64.0	12.0
1398	HFD	hNAG-1	25	8.0	32.0	16.0	20.0	32.0	16.0

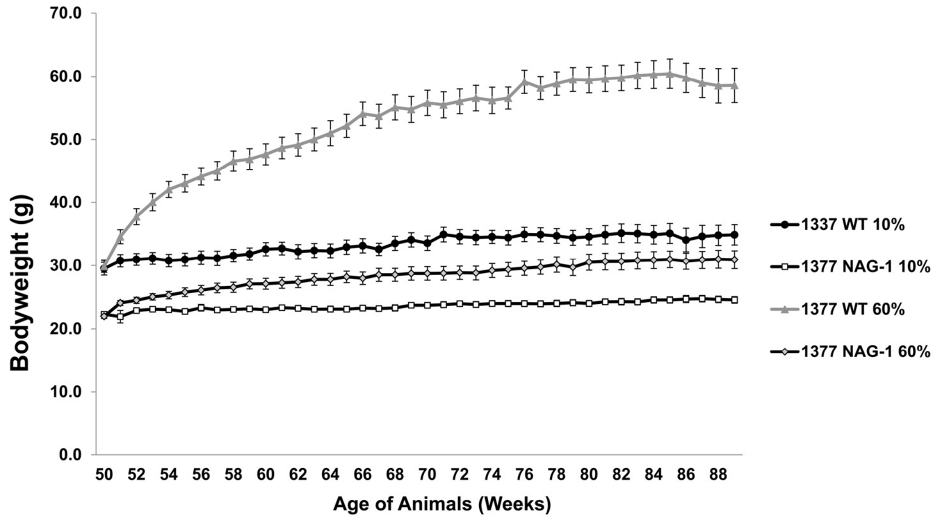

Figure S1. Body weights of mice (line 1377) over the course of the study. Female *hNAG-1* and WT mice were fed LFD or HFD at 50 wk old. Body weight change was recorded weekly for 40 wk long until mice are 90 wk old. The growth curves of WT and *hNAG-1* differed significantly on the LFD and HFD. The p-values for these differences were <0.0001.

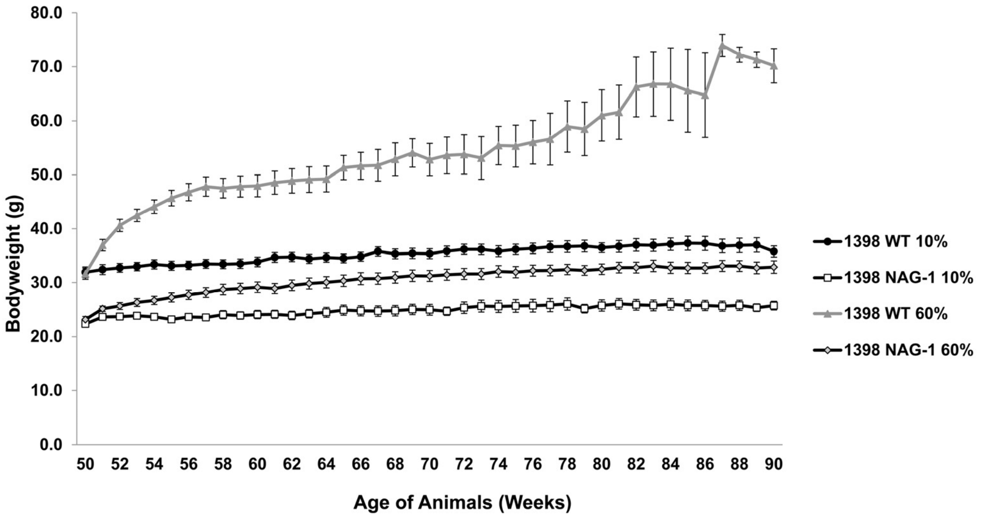

Figure S2. Body weights of mice (line 1398) over the course of the study. Female *hNAG-1* and WT mice were fed LFD or HFD at 50 wk old. Body weight change was recorded weekly for 40 wk long until mice are 90 wk old. The growth curves of WT and *hNAG-1* differed significantly on the LFD and HFD. The p-values for these differences were <0.0001.

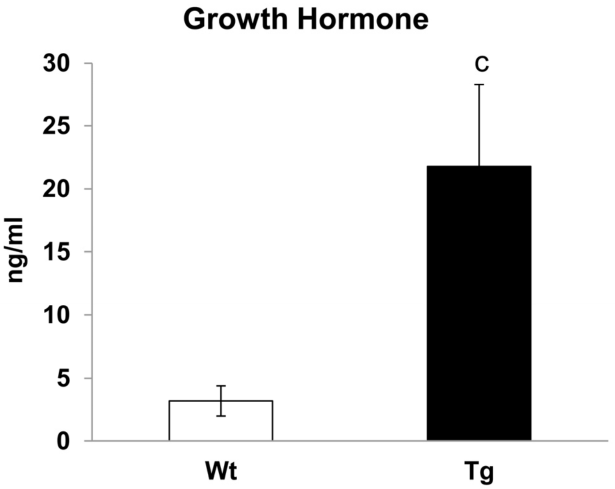

Figure S3. Serum growth hormone level in young *hNAG-1* and WT mice. Growth hormone was determined by ELISA in 20 wk old female 1398 mice. n=5~6/group. Data are presented as mean ± SE. c, p<0.001.

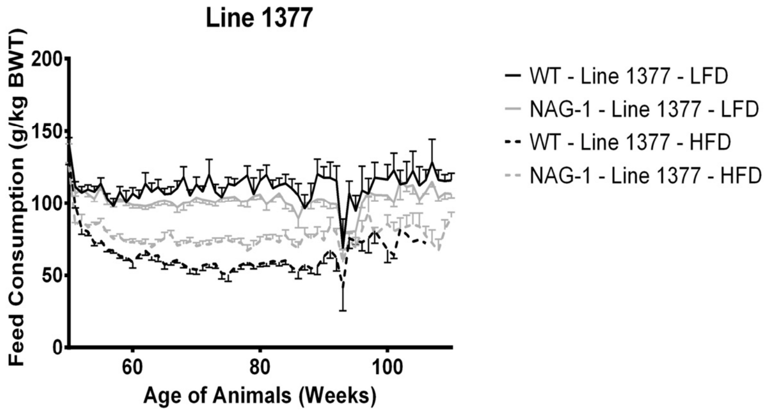

Figure S4. Food consumption in line 1377 mice. Female *hNAG-1* and WT mice were fed LFD or HFD at 50 wk old. Body weight change was recorded weekly for 40 wk long until mice are 90 wk old. Food consumption on the LFD was not significantly different between WT and *hNAG-1* for the 1377 line. Food consumption on the HFD was sporadically significantly different between WT and *hNAG-1* mice.

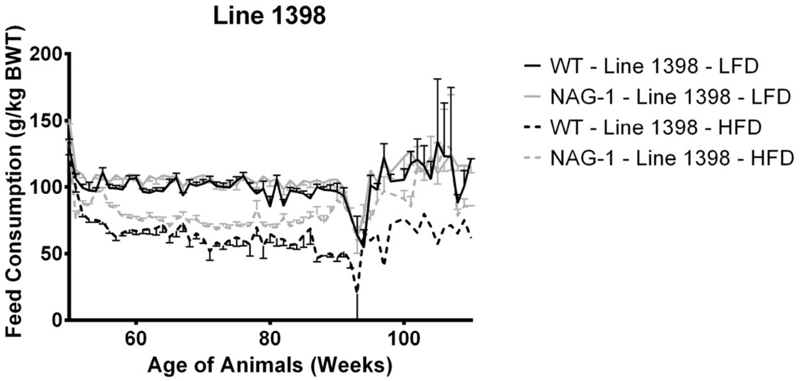

Figure S5. Food consumption in line 1378 mice. Female *hNAG-1* and WT mice were fed LFD or HFD at 50 wk old. Body weight change was recorded weekly for 40 wk long until mice are 90 wk old. Food consumption on the LFD was not significantly different between WT and *hNAG-1* for the 1398 line. Food consumption on the HFD was sporadically significantly different between WT and *hNAG-1* mice.

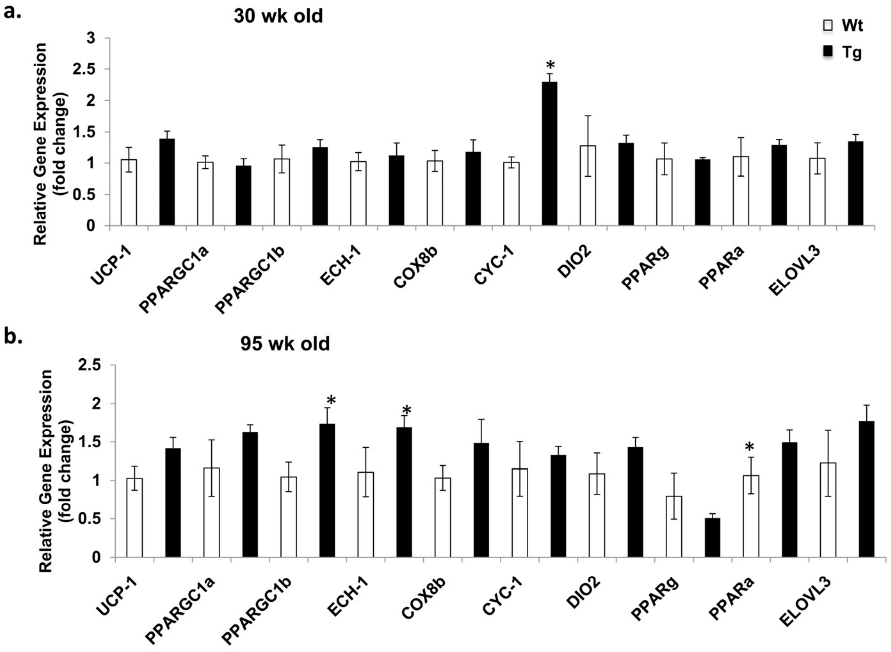

Figure S6. Expression of representative thermogenesis genes in BAT of 30 wk and 95 wk old mice in line 1398. Total RNA from BAT of female *hNAG-1* or WT mice on regular diet were extracted. The expression of representative genes of thermogenesis pathways in BAT was determined by *qRT-PCR* in 30 wk (**a**) and 95 wk (**b**) old animals. n=6/group. Data are presented as mean ± SE. *, p<0.05.

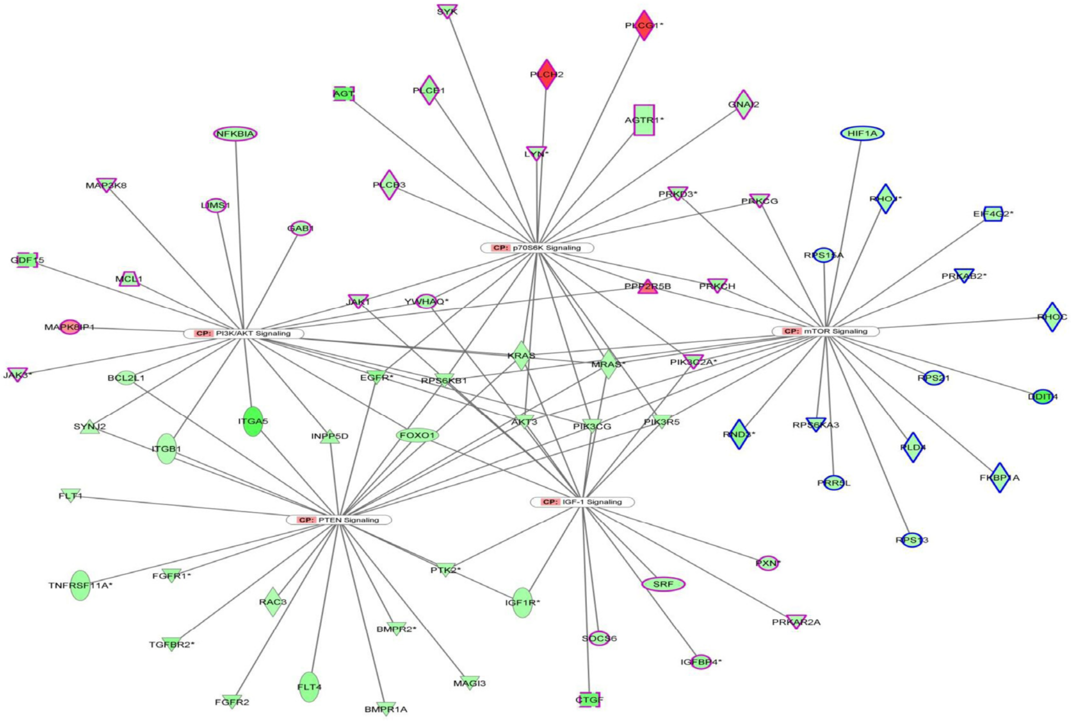

Figure S7. Gene-expression regulatory networks of directly connected signaling in *hNAG-1* mice. Gene-expression networks were analyzed by IPA network analysis. The relations between the genes were inferred from the relationships known in the scientific literature using data-mining Ingenuity software. Each node represents a gene; red color denotes over-expressed genes; green color denotes down-expressed genes. The colors intensity appears according to the related expression level by fold change. Connections indicate direct regulatory interactions.
